# AdNut study: effectiveness of a high calorie and protein oral nutritional supplement with β-hydroxy-β-methylbutyrate in an older malnourished population in usual clinical practice

**DOI:** 10.1007/s41999-018-0109-4

**Published:** 2018-11-16

**Authors:** D. A. de Luis, O. Izaola, L. López, B. Blanco, C. A. Colato, O. J. Kelly, R. Sanz, AdNut Group, Juan Ángel Hernández Bayo, Santiago Marcos Olivares, Francisco Villazón González, Patricia Sorribes Carreras, Virginia Mazoteras Muñoz, Alejandro Sanz, Diana Boj, Nuria Fernández Martínez, José María Tirado Moliner, Juan Ignacio Ramos-Clemente Romero, Cristóbal López Rodríguez, Mercedes Marco Martín, María Solange Amor Andrés, Miguel Araújo Ordóñez, Juan Antonio Herrera Tejedor, Lorena Salanova Chilet, Mercedes Vázquez Gutiérrez, Carmen Navarro Ortiz, Antonio Arroyo Sebastián, Roberto Hurtado García, Naiara Fernández Gutiérrez, Luis Alfonso Urquijo Hieyte, Elena Hervas Abad, Alicia Calleja Fernández, Francisco Manuel Suárez García, Eduardo Polo Marques, María Ángeles Penacho Lázaro, Arbis Acuña Carrazana, Carlos Pardo Ruiz, Carmina Wanden-Berghe Lozano, Javier González Nubla, Begoña Váquez Vizcaíno, Eva Rosell Vivancos, María Consuelo Martínez Burgui, José Vicente Raga Casasus, Begoña Ruiz Aguirre, Natalia Basy Iza, María del Carmen Zaballos Bautista, Luisa María Muñoz Salvador, Alfonso Carlos Aguirre Palacios, Sergio Niño Bernal, Eric Campello García, Nieves Pérez Climent, María Amparo Rodríguez Piñera, Julio José Lambea Sorrosal, María Argente Pla, Francisco Tarazona Santaballbina

**Affiliations:** 1 Centro de Investigación de Endocrinología y Nutrición Clínica Facultad de Medicina, Servicio de Endocrinología y Nutrición Hospital Clínico Valladolid, Av. Ramón y Cajal, 3, 47003 Valladolid, Spain; 2 Hospital San Juan, Alicante, Spain; 3 Hospital de Elda, Alicante, Spain; 4 Residencia AMMA el Balconcillo, Guadalajara, Spain; 5 Abbott Nutrition, Columbus, OH USA; 6 Abbott Nutrition, Madrid, Spain

**Keywords:** Malnutrition, Enteral nutrition, Safety, Nutritional status, Health related quality of life, Activities of daily living

## Abstract

**Objective:**

To evaluate the effectiveness of a high calorie and protein, β-hydroxy-β-methylbutyrate containing oral nutritional supplement (HP-HMB-ONS), on nutritional status, activities of daily living and quality of life (QoL) in old malnourished subjects.

**Methods:**

We conducted an observational, prospective, open label, multicenter study. Participants were > 65 years, undernourished or at nutritional risk [Nutrition Risk Score (NRS) ≥ 3] and had been included on an ONS (HP-HMB-ONS twice daily for 12 weeks) per standard of care. Visits at baseline (V1), 6 weeks (V2) and 12 weeks (V3) were performed. The primary endpoints were gain of body weight, change in body mass index (BMI) and NRS 2002 index. Data from QoL (EQ-5D-3L) and activities of daily living (Katz index) were also collected.

**Results:**

A total of 235 participants were included in the study. Of these 148 took at least a 75% of the HP-HMB-ONS and were included in the analysis (per protocol); median age was 80.0 (SD:8.3) years, 65.5% (*n* = 97) were female, 67.6% (*n* = 100) had 2 or more diseases. At V3, a statistically significant increase in weight (2.1 kg; SD: 3.8) (*p* < 0.001) and BMI (0.8 kg/m^2^; SD: 1.45) were found compared to V1, whereas NRS 2002 values decreased by 0.9 (SD: 1.2). A significant (*p* < 0.001) improvement in Katz index (mean change = 0.3; SD:1.4) and EQ-5D scoring (mean change = 0.5; SD:1.9) compared to V1, were also reported.

**Conclusions:**

The results suggest that administration of a HP-HMB-ONS improve the nutritional status and may led to a significant improvement in patients’ activities of daily living and QoL, independent of baseline BMI.

## Introduction

Malnutrition, strictly defined as a deficiency, excess or imbalance of energy, protein and other nutrients, is one of the most relevant, but under-recognized, conditions that adversely affects elderly people. The causes of malnutrition are many but several social, psychological or economic factors may compromise older people’s dietary intake and lead to nutritional deficiencies and malnutrition, together with the presence of acute and/or chronic conditions [1]. For the purpose of this study, we define malnutrition as an energy and nutrient insufficiency or deficiency that can be short (several days) or long (several months) in duration.

Malnutrition has serious consequences for individuals, causing a decline in functional status and worsening of existing medical issues, and an increased risk of mortality and morbidity [1]. Malnutrition is also associated with an increased risk of respiratory and cardiac problems, infections, deep venous thrombosis pressure ulcers, delayed wound healing, peri-operative mortality, multi-organ failure and immune dysfunction [2]. Moreover, a worsening of clinical outcomes in malnourished patients has been shown to be associated with a deterioration in quality of life (QoL) [3, 4] and longer hospital stays (LOS) [5, 6].

Many tools have been developed to screen for malnutrition in elderly patients, such as the Mini Nutritional Assessment (MNA) [7, 8], Nutrition Risk Score (NRS) [9], Malnutrition Universal Screening Tool (MUST) [10], Malnutrition Screening Tool (MST) [11], the Geriatric Nutritional Risk Index (GNRI) [12], as well as anthropometric and biochemical parameters. However, the prevalence of malnutrition varies according to the population studied and the tool used.

Malnutrition in the older population seems to be a global issue and Spain is no exception. A study performed in Spain showed that hospitalized patients have a high prevalence of malnutrition (23.9%) or were at risk of malnutrition (50.2%) [14]. Additionally, a prospective study using hospital admissions data over 24 h found an overall rate of undernutrition of 47.3%, raising to 54.2% in patients older than 65 years [15]. In 2012, a nationwide, cross-sectional, observational, Spanish multicenter study observed a prevalence of hospital malnutrition, according to NRS-2002, in 23.7% of patient [6].

Considering the high-prevalence and adverse consequences of malnutrition, the early identification of malnourished subjects and effective treatment is necessary. The majority of patients with or at risk of malnutrition, can be managed in several ways: (1) dietary counselling as indicated in clinical guidelines [16–19], (2) increase dietary consumption of food, or (3) using convenient oral nutritional supplements (ONS).

ONS utilization has been consistently associated with patient and clinical benefits, such as increase in body weight (weighted mean difference 2.25 kg; 95% CI 1.7–2.7) and muscle strength, functional improvements (walking distances and activities of daily living), immune function and QoL. In addition, clinical trials on ONS use conducted in hospital and community settings showed a reduction of complications associated with malnutrition (RR 0.44; 95% CI 0.3–0.6), and lower mortality rates in comparison with routine care (OR 0.7; 95% CI 0.5–0.9) [20].

In particular, a recent randomized controlled trial (NOURISH Study) showed that the early administration (within 72 h of hospitalization) of a high-protein ONS containing β-hydroxy-β-methylbutyrate (HP-HMB-ONS), in addition to standard of care, was associated with a decreased risk of mortality and improved nutritional status at 90-days post discharge [21]. However, to the best of our knowledge, the impact of HP-HMB-ONS on the quality of life of older malnourished patients [Nutritional risk scale (NRS) ≥ 3] living either in a nursing home or in the community, in routine clinical practice, has not yet been investigated in the Spanish setting.

In this light, the aim of the present study was to evaluate the effects of a HP-HMB-ONS (Ensure^®^ Plus Advance, Zwolle, Netherlands), on nutritional status, activities of daily living and QoL in older subject either living in a nursing home or receiving outpatient care in the Spanish setting.

## Methodology

### Design

We conducted an observational, prospective, multicenter study in the Spanish healthcare setting.

### Study population

Two different populations of patients living in nursing home and community older Spanish outpatients were recruited for the study.

Participants meeting the following inclusion criteria were eligible for the study: (1) 65 years of age or older; (2) being capable of giving informed consent; (3) being malnourished or at nutritional risk [(NRS) ≥ 3; BMI < 30 kg/m^2^]; (4) being under the supervision of an health care professional due to the malnutrition status; (5) having been included in an ONS plan (consisting in the administration of HP-HMB-ONS twice daily) at least 7 days prior to study enrollment; (6) living in a nursing home or receiving outpatient care.

The following exclusion criteria were set: (1) Subjects with renal or liver failure (defined as glomerular filtration rate GFR < 60 ml/min or serum aspartate aminotransferase [AST]/alanine aminotransferase [ALT] at least 3 times above the upper limit of normal); (2) Subjects diagnosed with type 1 or type 2 diabetes and/or on hypoglycemic agents or injectable insulin.

### Measurements

#### Nutritional status

Nutritional status of participants was assessed from baseline to visit 3 (12-weeks) by gain of body weight (kg), change in body mass index (BMI), and NRS 2002 (Score 0–7; NRS 2002 ≥ 3 indicates risk of malnutrition or malnutrition).

#### Functionality and quality of life

The Katz Index [26] was used to assess individual’s ability to perform 6 activities of daily living (ADL): feeding, toileting, bathing, dressing, continence, and transferring (0 = complete dependence to 6 = independence).

The EQ-5D-3L was used to evaluate QoL [27]. It is composed of two sections—the descriptive section (check a box) and a visual analogue scale (VAS) section (mark on a line). The EQ-5D-3L descriptive section contains 5 dimensions: mobility, self-care, usual activities, pain/discomfort and anxiety/depression. Each dimension then has 3 possibilities: no problems, some problems, extreme problems (each dimension 0 = best QoL to 3 = worst QoL; maximum total score: 15). The VAS section is used to obtain the participants self-reported health on a 20-cm vertical line, with the endpoints labelled ‘the best health you can imagine’ and ‘the worst health you can imagine’.

#### Safety

Adverse events (AE) defined as worsening of a pre-existing disease or condition, whether related or not to the treatment administered, we collected throughout the entire duration of the study.

### Data collection

Participants were evaluated, at 3 study visits, over a period of 12 weeks: at baseline (Visit 1; V1), at 6 weeks (Visit 2; V2) and at 12 weeks (Visit 3; V3). At baseline (V1), demographic data, recent blood chemistry records, nutritional status, QoL and functionality were collected. At V2 (study midpoint) and V3 (study completion), in addition to the measures collected at V1, nutritional status and functionality were collected. If the investigator decided to withdraw a participant from the study before completion (12 weeks), the reasons were recorded.

### Ethical considerations

All participants or legal representatives of participants signed the informed consent form. Data of all participants were recorded without including their personal details to maintain participant confidentiality. This post-marketing observational study was approved by the Clinical Research Ethics Committee of the Hospital Clinico Universitario de Valladolid.

### Statistical analysis

The data analysis for this paper was generated using SAS software, Version 9.3 for Windows. Copyright © 2016 SAS Institute Inc. SAS and all other SAS Institute Inc. product or service names are registered trademarks or trademarks of SAS Institute Inc., Cary, NC, USA. For continuous variables, mean and standard deviation (SD), values were calculated. The Shapiro–Wilks test was used to determine the normal distribution of the sample. For qualitative variables, absolute or relative frequencies were calculated. To analyze baseline differences between community living and institutionalized participants, Chi-square test or Fisher exact tests were performed. To analyze differences over time for body weight, BMI, NRS, ADL and QoL, Wilcoxon signed-rank test was performed.

To evaluate the effect of baseline BMI status (BMI ≤ 20 vs. BMI > 20 kg/m^2^) on other nutritional indicators in addition to functionality and QoL variables (Katz index and EQ-5D), a subgroup analysis was conducted, using a mixed-effects logistic regression model.

## Results

### Participant characteristics

A total of 235 participants were recruited. Of these, 2012 (90.2) consumed at least one fraction of the HP-HMB-ONS and constituted the modified intention-to-treat population (mITT). Of this population, 148 (62.9%) completed the 12 weeks follow-up having reported consuming at least 75% of the HP-HMB-ONS prescription (twice daily for 12 weeks) and were included in the as-treated (per protocol) analysis. A total of 5 (3.4%) participants withdrew early from the study; voluntarily (*n* = 1, 0.7%), medical decision (*n* = 1, 0.68%), lost to follow-up (*n* = 2, 1.4%) and 1 (0.7%) participant died during the study.

Women accounted for 65.5% (*n* = 97) of the per protocol sample, and the median age, for all participants, was 80.0 years [interquartile range (IQR) 74–86.5]. All participants were at risk of malnutrition with a mean (± SD) weight at baseline of 52.2 ± 10.8 kg. Mean body mass index (BMI) was 20.5 ± 3.3 kg/m^2^ at baseline. The majority of participants were living at home (*n* = 97, 65.5%) with the remainder (*n* = 51, 34.5%) residing in Spanish nursing homes. Comorbidities included: 55.4% (*n* = 82) with hypertension, 46.5% (*n* = 33) with a neurodegenerative disease, 29.5% (*n* = 43) had cancer, and up to 18.2% (*n* = 27) had dyslipidemia. Notably, most participants included (67.6%; *n* = 100) had multiple comorbidities at the time of the study. Table 1 shows baseline characteristics for study population: miTT population, per protocol population (community living and institutionalized participants). The results of the comparison of baseline characteristics of per protocol population showed statistically significant differences in age between community living and institutionalized participants.

**Table 1:** Baseline demographic characteristics

Characteristics (*N* = 148)	Total participants (mITT)^a^ (*n* = 212)	Total participants (per protocol)^b^ (*n* = 148; 63.0%)	Community living participants (*n* = 97; 65.5%)	Institutionalized participants (*n* = 51; 34.5%)	*p*
Gender [*n* (%)]					
Women	134 (63.2%)	97 (65.6%)	66 (68.0%)	31 (60.8%)	0.241*
Men	78 (36.8%)	51 (34.4%)	31 (31.9%)	20 (39.2%)	
Age (years) [mean ± SD]	80.7 ± 8.24	80 ± 8.3	78.4 ± 8.3	82.9 ± 7.6	0.0025**
Weight (kg) (mean ± SD)	52.9 ± 10.5	52.2 ± 10.9	52.9 ± 10.6	50.7 ± 11.4	0.1341**
BMI (kg/m^2^) (mean ± SD)	20.6 ± 3.3	20.5 ± 3.3	20.7 ± 3.2	20.1 ± 3.5	0.1846**
Comorbidities [*n* (%)]					
Hypertension	119 (56.1%)	82 (55.4%)	51 (52.6%)	31 (60.8%)	0.34*
Cardiovascular disease	74 (34.9%)	47 (31.7%)	28 (28.9%)	19 (37.3%)	0.297*
Cancer	60 (28.3%)	43 (29.5%)	33 (34.0%)	10 (19.6%)	0.066*
Dyslipidemia	39 (18.4%)	27 (18.2%)	16 (16.5%)	11 (21.6%)	0.448*
Kidney disease	10 (4.72%)	4 (2.7%)	2 (2.1%)	2 (3.9%)	0.608***
Hepatobiliary disease	7 (3.30%)	4 (2.7%)	4 (4.1%)	0 (0.0%)	0.299***
Other^c^	109 (51.4%)	71 (48.0%)	41 (42.3%)	30 (58.8%)	0.055*
Weight loss in the last 3 months [*n* (%)]	196 (92.4%)	139 (93.92%)	93 (95.9%)	46 (30.2%)	0.276***
Reduction of food intake [*n* (%)]	173 (81.6%)	119 (80.41%)	81 (83.5%)	38 (74.5%)	0.19*

*BMI* body mass index, *IQR* interquartile range, *SD* standard deviation

*Chi-square test

**Kruskal–Wallis equality-of-populations rank test

***Fisher’s exact test

^a^Study participants that has received at least one portion of the HP-HMB-ONS

^b^Study participants who met the eligibility criteria and who had consumed at least 75% of HP-HMB-ONS

^c^Including neurodegenerative and lung disease

All study subjects who received at least one serving of HP-HMB-ONS (*n* = 212) were included in the intention to treat population in addition to the safety analysis by summarising adverse events. For this analysis, the median age was 81 years old (IQR 74 – 87) with 63.2% being female. The proportion of participants living at home was 60%. The majority of participants included had hypertension 56.1% (*n* = 119), 34.9% (*n* = 74) had cardiovascular disease and cancer was present in 28.3% (*n* = 60) of participants.

### Effectiveness of HP-HMB-ONS on participant’s nutritional status

All three nutrition-related endpoints (body weight, BMI and NRS 2002) assessed in the per protocol population (*n* = 148) were significantly improved by the end of the study (V3; 12 weeks). From baseline (V1) to the midpoint (V2) mean weight increased significantly (*p* < 0.001) by 1.6 ± 2.7 kg (mean ± SD), and from V1 to V3 (end of the study) (*p* < 0.001) increased by 2.1 ± 3.8 kg. As expected with weight increases, BMI also significantly increased by 0.6 ± 1 kg/m^2^ between V1 and V2, and by 0.8 ± 1.5 kg/m^2^ between V1 and V3.

**Fig. 1:**
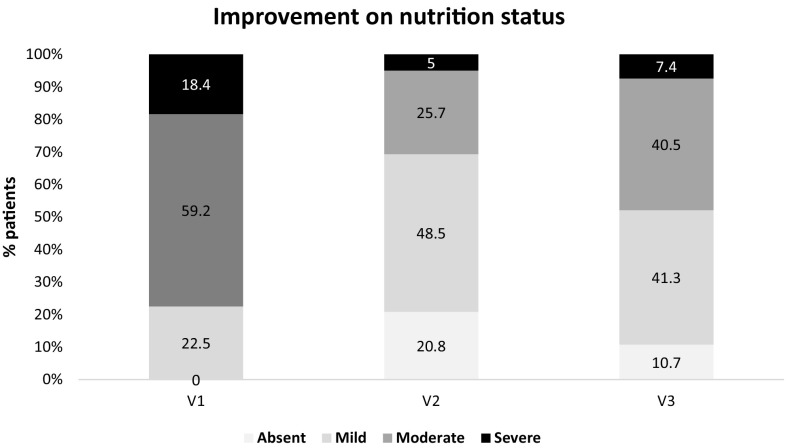
Improvement on nutritional status from V1 to V3 according to NRS 2002. Absent, normal nutritional status; mild, weight loss 45% in 3 months or Food intake below 50–75% of normal requirement in preceding week; moderate, weight loss 45% in 2 months or BMI 18.5–20.5 + impaired general condition or food intake 25–60% of normal requirement in preceding week; severe, weight loss 45% in 1 month (415% in 3 months) or BMI o18.5 + impaired general condition or food intake 0–25% of normal requirement in preceding week; V1, Baseline; V2, Visit at 6 weeks; V3, Visit at 12 weeks

Although to be eligible all participants had a NRS-2002 score ≥ 3, positive changes in NRS-2002 score were also observed. Between V1 and V2 the NRS-2002 score decreased by 0.6 ± 1 (*p* < 0.001), and by 0.9 ± 1.2 (p = 0.002) between V1 and V3. Combined, these results show that nutritional status recovered after 6 and 12 weeks of HP-HMB-ONS (Table 2, Fig. 1).

**Table 2:** Variables at the 3 visits and changes from Visit 1

Variable	Mean (SD)		Median (IQR)		Difference from V1 mean (SD)		Difference from V1 median (IQR)		*p*
Weight (kg)									
V1	52.16 (10.85)		51 (45–58)		NA		NA		NA
V2	53.64 (10.9)		52.3 (47–58)		− 1.61 (2.7)		− 1 (− 2.0 to − 0.4)		< 0.001*
V3	54.29 (11.0)		53 (47–60)		− 2.14 (3.8)		− 2 (− 3.4 to − 1)		< 0.001*
BMI (kg/m^2^)									
V1	20.51 (3.3)		20 (18.1–22.2)		NA		NA		NA
V2	21.12 (3.3)		20.5 (18.9–22.9)		− 0.62 (1.0)		− 0.42 (− 0.81 to – 0.12)		< 0.001*
V3	21.34 (3.3)		20.89 (19.2–23.2)		− 0.8 (1.5)		− 0.8 (− 1.33 to − 0.39)		< 0.001*
NRS 2002									
V1	3.94 (0.9)		4 (3–5)		NA		NA		NA
V2	3.3 (1.2)		3 (3–4)		0.6 (1)		0 (0–1)		< 0.001*
V3	3.1 (1.2)		3 (2–4)		0.9 (1.2)		1 (0–2)		< 0.001*
Activities of daily living [Katz index of independence in activities of daily living (Katz score)]									
V1		2.8 (2.5)		2 (0–6)		NA		NA	NA
V2		3.0 (2.4)		2 (1–6)		− 0.2 (1.0)		0 (0–0)	0.0088
V3		3.0 (2.5)		3 (1–6)		− 0.3 (1.4)		0 (0–0)	0.0194
Quality of life									
EQ-5D-3L (VAS)									
V1		49.9 (18.5)		50 (40–60)		NA		NA	NA
V2		57.5 (16.6)		60 (50–70)		− 7.4 (12.2)		− 5 (− 10 to 0)	< 0.001*
V3		60.9 (18.4)		60 (50–75)		− 10.9 (14.8)		− 10 (− 20 to 0)	< 0.001*
EQ-5D-3L (total)									
V1		9.7 (2.3)		10 (8–11)		NA		NA	NA
V2		9.4 (2.4)		9 (8–11)		0.4 (1.5)		0 (0–1)	< 0.001*
V3		9.3 (2.6)		3 (2–4)		0.5 (1.9)		0 (0–1)	< 0.001*

*BMI* body mass index, *EQ-5D* EuroQol-5D, *IQR* interquartile range, *NA* not applicable, *NRS* nutritional risk screening, *SD* standard deviation, *VAS* visual analog scale, *V1* baseline, *V2* visit at 6 weeks, *V3* visit at 12 weeks

*Statistically significant difference

### Effectiveness of HP-HMB-ONS on participant’s quality of life (QoL) and functionality

In general, supplementation with HP-HMB-ONS, led to a significant improvement in ADL and QoL scores. The mean Katz Index of Independence in Activities of Daily Living (Katz score), which evaluates the degree of a patient’s dependence on others to perform ADL, rose by 0.2 ± 0.2 points (*p* < 0.001) between baseline (V1) and the study midpoint (V2) and by 0.3 ± 1.4 points (*p* < 0.001) between V1 and the end of the study (V3), showing patients became less dependent, and possibly more independent, by the end of the study (Table 2). The individual scores for each ADL are outlined in Table 2. Improvements were seen for all activities included in the Katz score from V1 to V3: bathing (V1 32.4% vs. V3 34.5%), dressing (V1 41.2% vs. V3 36.6%), toileting (V1 43.2% vs. V3 46.6%), transferring (V1 47.3% vs. V3 51.3%), continence (V1 50.7% vs. V3 52.7%) and feeding (V1 62.8% vs. V3 72.3%).

Overall mean QoL, as measured by EQ-5D, significantly improved from baseline V1 to V2 by 0.4 ± 1.5 points (*p* < 0.001) and from V1 to V3 by 0.5 ± 1.9 points (*p* < 0.001). The VAS scores were increased significantly at both V2 and V3 by 7.4 ± 12.2 points (*p* < 0.001) and 10.9 ± 14.8 points (*p* < 0.001), respectively, suggesting that improving nutritional status results in better participant-perceived health status (Table 2). For each EQ-5D dimension there were some differences to treatment response, however, for those participants that reported having ‘some problems’ the results were relatively consistent from study visit to study visit: mobility (V1 62.2% vs. V3 56.1%), self-care (V1 43.2% vs. V3 37.2%), usual activities (V1 45.3% vs. V3 41.2%), pain/discomfort (V1 66.9% vs. V3 49.3%) and anxiety/depression (V1 56.8% vs. V3 46.6%), see Table 3.

**Table 3:** Percentage of subjects which reported “some problems” on the health status dimensions

Responses (total *n* = 148)	V1 [*n* (%)]	V2 [*n* (%)]	V3 [*n* (%)]	Diff V1 –V2	Diff V2 –V3	Diff V1 –V3
Mobility						
I have no problems in walking about.	29 (19.6)	31 (21.0)	36 (24.3)	↑	↑	↑
I have some problems in walking about	92 (62.2)	92 (62.2)	83 (56.1)	–	↓	↓
I am confined to bed	26 (17.6)	24 (16.2)	28 (18.9)	↓	↑	↑
Missing	1 (0.7)	1 (0.7)	1 (0.7)			
Self-care						
I have no problems with self-care	38 (25.7)	43 (29.1)	47 (31.8)	↑	↑	↑
I have some problems washing or dressing myself	64 (43.2)	61 (41.2)	55 (37.2)	↓	↓	↓
I am unable to wash or dress myself	45 (30.4)	43 (29.1)	45 (30.4)	↓	↑	–
Missing	1 (0.7)	1 (0.7)	1 (0.7)			
Usual activities						
I have no problems with performing my usual activities	23 (15.5)	31 (21.0)	31 (21.0)	↑	–	↑
I have some problems with performing my usual activities	67 (45.3)	65 (43.9)	61 (41.2)	↓	↓	↓
I am unable to perform my usual activities	57 (38.5)	51 (34.5)	55 (37.2)	↓	↑	↓
Missing	1 (0.7)	1 (0.7)	1 (0.7)			
Pain/discomfort						
I have no pain or discomfort	41 (27.7)	54 (36.5)	66 (44.6)	↑	↑	↑
I have moderate pain or discomfort	99 (66.9)	85 (57.4)	73 (49.3)	↓	↓	↓
I have extreme pain or discomfort	7 (4.7)	8 (5.4)	8 (5.4)	↑	–	↑
Missing	1 (0.7)	1 (0.7)	1 (0.7)			
Anxiety/depression						
I am not anxious or depressed	52 (35.1)	68 (46.1)	72 (48.7)	↑	↑	↑
I am moderately anxious or depressed	84 (56.8)	71 (48.0)	69 (46.6)	↓	↓	↓
I am extremely anxious or depressed	11 (7.4)	8 (5.4)	6 (4.1)	↓	↓	↓
Missing	1 (0.7)	1 (0.7)	1 (0.7)			

### Effects of baseline BMI status on outcomes

At baseline 74 (50.3%) participants had a BMI ≤ 20 kg/m^2^, whereas 73 (49.7%) participants had a BMI of > 20 kg/m^2^, with one of the participants with some missing BMI data. Therefore, the mixed-effects logistic regression model was conducted with data from 147, of the 148 evaluable, participants.

No association was observed according to the mixed-effects logistic regression between baseline BMI (BMI ≤ 20 kg/m^2^ vs. BMI > 20 kg/m^2^) and other nutritional (weight loss, NRS), functional (Katz index) or QoL variables (EQ-5D) (P > 0.05), indicating that nutritional, functional and QoL improvements were independent of baseline BMI values (Table 4).

**Table 4:** Results of mixed-effects logistic regression model of BMI and functional and quality of life outcomes

Effect	Num DF	*F* value	*p* value
Weight			
Baseline BMI	1	75.5	< 0.0001
Visit	2	35.5	< 0.0001
Baseline BMI × visit	2	2.9	0.0609
NRS 2002			
Baseline BMI	1	8.9	0.0033
Visit	2	30.2	< 0.0001
Baseline BMI × visit	2	2.7	0.0744
Katz index			
Baseline BMI	1	0.2	0.6838
Visit	2	32.5	< 0.0001
Baseline BMI × visit	2	0.6	0.5536
Quality of Life (Total score EQ-5D-3L)			
Baseline BMI	1	2.6	0.1082
Visit	2	5.8	0.0039
Baseline BMI × visit	2	0.5	0.6089

*DF* degrees of freedom, *Baseline BMI × visit* interaction between baseline BMI and changes observed during follow-up visits

### Product safety

14.6% (*n* = 31) of participants included in the safety analysis (*N* = 212), reported at least one adverse event (AE), whereas 7.1% (*n* = 15) had a severe adverse effect (SAE). Only 1.4% (*n* = 3) of AE were possibly or probably related to the treatment, all of them were mild or moderate gastrointestinal complications (one participants had diarrhea, one case of nausea and one of vomiting). None of the SAE were HP-HMB-ONS related.

## Discussion

Malnutrition is an important factor contributing to poorer health and functional impairment. In addition, the impact of nutritional status on physical and psychological well-being may be especially important in the older subjects, as they are more likely than younger adults to have a poorer nutritional status and are at a higher risk for disease-related malnutrition [28].

The results of the study suggested that a high-protein ONS with HMB and vitamin D, was effective at improving nutritional status, which may lead to increases in ADL and QoL scores, in both older Spanish institutionalized and free-living population. These results are in agreement with a previous study conducted in Spain, which demonstrated that the addition of a HP-HMB-ONS improves the nutritional status of institutionalized older Spanish population [29]. Other studies [30–32], have reported the improvement in nutritional status of community-dwelling, institutionalized and free living malnourished elderly population by prescribing ONS to fill the energy and nutrient deficits.

A meta-analysis of 55 studies on protein and energy supplementation in older people showed that for undernourished participants in short-term care facilities, use of ONS was associated with fewer complications and reduced mortality [33]. Stratton et al. showed that the use of oral nutritional supplements improved protein and energy intake by restoring appetite and increasing the feeling of well-being [34]. In addition, a comprehensive systematic review of 84 studies in free living outpatients showed that ONS use increased total energy intake, however, > 50% of the daily energy was provided by the ONS [35], suggesting appetite is greatly reduced in older outpatients. Interestingly this systematic review showed that participants’ with a BMI < 20 kg/m^2^ were more likely to see improvements compared to those with a BMI > 20 kg/m^2^. Furthermore, other reports show that it may be only feasible to measure the beneficial effects of nutritional supplements on undernourished participants (BMI ≤ 20 kg/m^2^) [1]. Conversely, this study showed that the improvement in weight gain with ONS was obtained independently of baseline BMI values. Thus, HP-HMB-ONS may be effective in improving participants’ nutritional status regardless of their initial weight of BMI.

HP-HMB-ONS, in this study, was well tolerated. Only 3 participants had mild or moderate gastrointestinal complications and no SAE was related to the study treatment. Therefore, HP-HMB-ONS is a safe and effective option when choosing a nutritional treatment for patients at malnutrition risk or even malnourished patients. More importantly, in line with previous studies [36], this study suggests that regular administration of beta-hydroxy-beta-methylbutyrate enriched supplements not only addresses patients’ nutritional needs, but also improves functionality and QoL. Improvements were seen in most of ADL and in the 5 dimensions of QoL, as well as in the self-perceived health status (as measured by a VAS).

Beyond its benefits in terms of nutritional status and QoL improvement, a recent study has shown that ONS in older hospitalized patients contributes to reducing both re-admission and hospital length of stay [37].

These results must be interpreted in the context of the study limitations, the main one being the lack a control group. However, considering that to be eligible, participants had to be undernourished or at nutritional risk (NRS ≥ 3), it was deemed unethical to deprive participants of a nutritional intervention. It must also be noted that community living and institutionalized participants might show different clinical and/or functional characteristics (e.g. strength, usual food intake, exercise level, etc.) that could influence the results. However, due to the lack of availability of data regarding the mentioned aspects, we were not able to rule out the possibility that some of these confounders may explain, at least in part, the results obtained and further studies are needed to elucidate these aspects. Finally, although we acknowledge that a comparison between institutionalized and community living participants might have helped clarifying whether one of the two groups might benefit the most from ONS, our study was not powered enough to detect statistically significant differences between the groups.

In conclusion, the study results suggest that a 12-week intervention with a HP-HBM-ONS may improve nutritional status, functionality and QoL in community older adults or living in nursing home in Spain. Notably, this improvement occurs independently of participants BMI baseline level.

HP-HBM-ONS may not only have the potential to enhance the nutritional condition of both community older adults or living in nursing home but also it may enable to increase their self-reliance in activities of daily living and achieve improved QoL.
